# Indications for Endoscopic Ultrasound-Guided Pancreatic Drainage: For Benign or Malignant Cases?

**DOI:** 10.1155/2018/8216109

**Published:** 2018-06-03

**Authors:** Daisuke Uchida, Hironari Kato, Yosuke Saragai, Saimon Takada, Sho Mizukawa, Shinichiro Muro, Yutaka Akimoto, Takeshi Tomoda, Kazuyuki Matsumoto, Shigeru Horiguchi, Hiroyuki Okada

**Affiliations:** Department of Gastroenterology, Okayama University Hospital, 2-5-1 Shikata-cho, Kita-ku, Okayama 700-8558, Japan

## Abstract

**Background and Aims:**

Recurrent pancreatitis associated with pancreatic strictures requires treatment with endoscopic retrograde pancreatography (ERP), but it is sometimes technically unsuccessful. Endoscopic ultrasound-guided pancreatic drainage (EUS-PD) was developed as an alternative to a surgical approach after failed ERP; however, the indications for EUS-PD are unclear. In this study, we evaluated the outcomes of EUS-PD and established the indications for EUS-PD.

**Methods:**

A total of 15 patients had indications for EUS-PD for recurrent pancreatitis due to pancreatic strictures. There were eight patients with benign pancreatic strictures and seven with malignant pancreatic strictures. The success rate, adverse events, and long-term outcomes were evaluated.

**Results:**

The technical success rates of benign and malignant strictures were 75% (6/8) and 100% (7/7), respectively, and clinical success was achieved in 100% (6/6) and 87.5% of cases (6/7), respectively. Rendezvous procedures were performed in two patients with benign strictures. The adverse event (AE) rate was 26.7% (4/15) and included cases of peritonitis, bleeding, and stent migration. Reinterventions were performed in three patients with benign strictures and two with malignant strictures.

**Conclusions:**

EUS-PD was an appropriate treatment for not only benign strictures but also malignant strictures with recurrent pancreatitis after failed ERP. However, the AE rate was high, and reinterventions were required in some cases during long-term follow-up. The indications for EUS-PD should be considered carefully, and careful follow-up is needed.

## 1. Introduction

Symptomatic pancreatic strictures are troubling problems associated with pancreatic diseases. Benign pancreatic strictures have various causes, such as chronic pancreatitis, anastomotic strictures after pancreaticoduodenectomy, and traumatic pancreatic injuries. Endoscopic retrograde pancreatography (ERP) and drainage are feasible treatments for benign strictures [1–3]. Recently, ERP in altered anatomy using a balloon-assisted enteroscope was developed, and anastomotic strictures after surgery can now be treated without surgical treatment [4]. Malignant pancreatic strictures with recurrent pancreatitis are rare but sometimes occur in cases with pancreatic head tumors. ERP is the standard approach for symptomatic malignant strictures, as with benign ones; however, some patients require a surgical approach [5–7]. Surgical treatment is a gold standard therapy for uncontrollable recurrent pancreatitis but is an invasive treatment with a high adverse event (AE) rate [8, 9].

Endoscopic ultrasound- (EUS-) guided pancreatic drainage (EUS-PD) was developed as an alternative to a surgical approach after failed ERP; however, the indications for EUS-PD remain controversial [10, 11]. It is a feasible treatment but is technically challenging, and the AE rate is relatively high [10, 12, 13]. In this study, we evaluated the outcomes of EUS-PD and considered the indications.

## 2. Materials and Methods

### 2.1. Patients

Fifteen patients with recurrent pancreatitis who were admitted to Okayama University Hospital from September 2012 to December 2017 were enrolled. Conventional ERP had been attempted in all patients, but drainage could not be achieved. EUS-PD was carried out after failed ERP and after obtaining written informed consent. There were nine patients with benign pancreatic strictures. Pancreatic drainage was required for strictures of pancreatodigestive anastomosis after pancreatoduodenectomy in six patients and chronic pancreatitis in two patients. In contrast, there were seven patients with malignant pancreatic strictures associated with pancreatic head tumors, six patients with pancreatic cancer, and one patient with cholangiocarcinoma (Table 1). All of them had distant metastasis and were not indicated for surgical treatments.

### 2.2. EUS-PD Procedure

**Figure 1:**
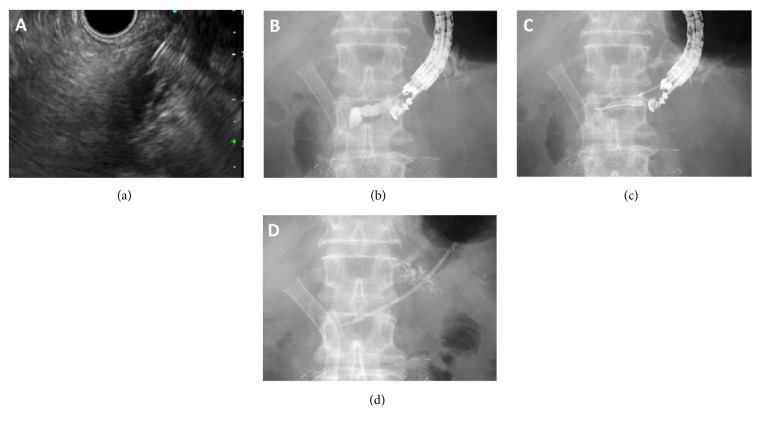
The EUS-PD procedure for a patient with obstructive pancreatitis due to pancreatic head cancer. (a) The pancreatic duct was punctured via the stomach with a 19-gauge needle under EUS guidance. (b) The pancreatogram was obtained by the injection of contrast agent. (c) A 0.025-inch guidewire was advanced into the pancreatic duct, and the tract was dilated using a long-tapered catheter or a diathermy catheter. (d) A 7-Fr plastic stent was inserted over the guidewire.

The EUS-PD procedure is shown in Figure 1. The dilated pancreatic duct was punctured anterogradely via the stomach by a 19-gauge needle under EUS guidance. The 0.025-inch guidewire (Visiglide2; Olympus, Tokyo, Japan) was advanced as far as possible. The puncture tract was then dilated by a long-tapered catheter (3.5Fr; PR-V220Q, Olympus, Tokyo, Japan). If this dilation failed, a diathermy catheter was used to dilate the tract. Finally, a 7-Fr plastic stent (Flexima; Boston Scientific Japan, Tokyo, or Advanix; Boston Scientific Japan, Tokyo, or Cotton-Leung; COOK Japan, Tokyo) was placed into the pancreatic duct. In patients with benign strictures, stents were placed across the papilla or at the anastomotic sites if feasible.

### 2.3. Definition of Success

Technical success was defined as the placement of a plastic stent into the pancreatic duct. Clinical success was defined as the improvement of symptoms and pancreatitis within a week.

## 3. Results

The patients' characteristics and outcomes are shown in Table 1. The technical success rate was 86.7% (13/15), and clinical success was achieved in 92.3% (12/13) of cases. The technical and clinical success rates of benign strictures were 75% (6/8) and 100% (6/6), respectively, and those of malignant strictures were 100% (7/7) and 85.7% (6/7), respectively. There were two technical failures in benign cases. In both patients, EUS-PD was attempted because anastomotic strictures developed after pancreaticoduodenectomy, but plastic stents could not be placed because of unsuccessful advancement of the guidewire after puncture (cases 1 and 2). They were treated with a percutaneous approach and ultimately improved. Clinical success was not achieved in one patient with malignant stricture (case 13). He underwent EUS-PD for obstructive pancreatitis with pancreatic head cancer and failed to regain the ability for oral intake until his death due to his poor general condition.

**Figure 2:**
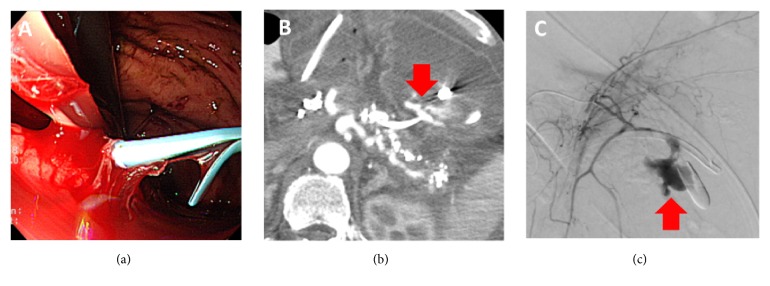
Bleeding occurred at the 371st day after EUS-PD in case 3. (a) Arterial bleeding from the transgastric puncture tract. (b) Contrast-enhanced computed tomography revealed extravasation into the stomach (arrow). (c) Interventional radiology revealed a pseudoaneurysm from the left gastric artery (arrow), and arterial embolization was performed.

The median follow-up period was 223 days (benign: 503 days, malignant: 116 days). AEs occurred in 4 cases (26.7%) during the follow-up period, including 3 benign cases and 1 malignant case. In the benign cases, bleeding, stent migration, and peritonitis occurred. Peritonitis occurred in case 8 the day after the procedure. He was suspected of having pancreatic juice leakage from the side holes of the plastic stent, and he required replacement of a plastic stent. Stent migration into the stomach was detected incidentally at the 97th day after the procedure in case 4. He was followed up with conservative treatment because he had no symptoms. Bleeding from the puncture tract occurred at the 371st day after EUS-PD in case 3 (Figure 2). In this case, a rendezvous procedure failed, and periodic stent replacement via the puncture tract was performed because of recurrent pancreatitis due to stent occlusion. He had a bleeding from puncture tract and required arterial embolization with interventional radiology. In the malignant cases, only one AE (peritonitis) occurred the day after the procedure; however, the patient's condition improved with conservative treatment (case 15).

Reinterventions were performed in four patients (cases 3, 5, 8, and 10). Cases 3 and 10 underwent stent replacement because of recurrent pancreatitis associated with stent occlusion (patency times: 54 and 224 days, respectively). All of them achieved technical and clinical success, but they still require periodic stent replacement. Case 5 underwent rendezvous procedure for benign stricture with chronic pancreatitis. He was also treated with transpapillary stent replacements at regular intervals. Case 8 developed peritonitis the day after EUS-PD, which might have been caused by pancreatic juice leakage via the puncture tract. The stent was then exchanged to a different type of plastic stent without side holes (Through Pass TYPE-IT; Gadelius Medical, Tokyo, Japan). After reintervention, the patient's peritonitis improved.

## 4. Discussion

Recurrent pancreatitis is a troubling problem associated with pancreatic disease and is caused by pancreatic stricture in most cases. There are various causes of stricture, such as chronic pancreatitis, anastomotic stenosis after surgery, and malignant tumor. A retrograde approach with ERP is most common treatment for these strictures [1–6]. However, ERP sometimes fails because of technical difficulties, and a percutaneous or surgical approach is required. EUS-PD was developed as an alternative treatment to these invasive approaches after failed ERP [10, 11, 15, 17, 18]. While this is an innovative and useful procedure, it remains technically challenging and is associated with a high AE rate. Tyberg et al. reported the findings of a multicenter retrospective study of EUS-PD [13]. They found a high success rate, although the AE rate was as high as 20%. Various AEs were noted, including severe ones that required surgical treatment. The study was a relatively large-scale study of 80 patients; however, no predictors of AE were identified. Oh et al. reported a high success rate of EUS-PD with a fully covered self-expandable metal stent, but the AE rate was also high in that study (Table 2) [14]. There are some other reports, but the indication for EUS-PD is still unclear [10, 12, 15, 19]. We evaluated our case series and considered the indications for EUS-PD.

Anastomotic stricture after failed balloon-assisted ERP is a candidate for EUS-PD. The recently developed balloon-assisted ERCP technique has allowed anastomotic pancreatic and biliary strictures after surgery to be treated without a percutaneous or surgical approach [4, 16, 20]. However, pancreatic anastomosis is often more difficult than biliary anastomosis. In our study, 6 of 15 patients underwent EUS-PD after failed balloon-assisted ERP. Two of these patients failed their procedure due to the operator's inexperience and required percutaneous treatments, while the other four achieved clinical success and benefitted significantly from EUS-PD.

We consider that obstructive pancreatitis associated with pancreatic tumor is also a candidate indication for EUS-PD. In our series, there were seven patients with malignant pancreatic strictures. All of them achieved technical success, and six of them achieved clinical success. Peritonitis occurred in case 15, but her condition improved with conservative treatment. Stent occlusion occurred in case 10 at the 224^th^ day after EUS-PD, and reintervention was performed. Recurrent pancreatitis did not occur due to periodic stent replacement after stent occlusion. AEs, including stent occlusion, were rarer in cases with malignant strictures (12.5%) than in those with benign strictures (37.5%), though not to a significant degree (P=0.12). This discrepancy might have been caused by the difference in the follow-up period (116 days and 503 days). EUS-PD may be feasible as a palliative treatment for patients with malignant strictures.

Chronic pancreatitis is the most common disease causing troubling pancreatic strictures. In this study, two patients with chronic pancreatitis underwent EUS-PD after failed ERP. Both achieved technical and clinical success, but bleeding from a fistula associated with pseudoaneurysm occurred at the 371st day after EUS-PD in case 3. He underwent periodic stent replacement because a rendezvous procedure failed due to pancreatic stones. Therefore, case 3 required long-term stent placement via the puncture tract. Pseudoaneurysm from the gastric artery might be induced by mechanical stimulation with long-term stent placement and inflammation of chronic pancreatitis. Kurihara et al. also reported the occurrence of aneurysms associated with the EUS-PD procedure in patients with recurrent pancreatitis [19]. Stent removal should be considered in cases with chronic pancreatitis. If stent removal is impossible, a surgical approach should be considered.

In our cases series, benign strictures requiring long-term stent placement might be predictors of AEs, such as bleeding or stent migration. However, this study is limited by the small number of patients and its retrospective design, and further prospective evaluations should be performed.

In conclusion, EUS-PD conferred benefits on patients with uncontrollable recurrent pancreatitis. In cases of benign strictures, especially with chronic pancreatitis, rendezvous procedures and eventual stent removal should be considered to avoid late adverse events. In cases with malignant strictures, a high success rate was achieved, and severe AEs did not occur. Therefore, EUS-PD is a promising approach as a palliative treatment for patients with malignant strictures. However, EUS-guided puncture procedures should be avoided in patients with resectable tumors, as there are risks of tumor cell dissemination.

EUS-PD is a feasible and safe approach but is still a relatively primitive procedure with a high AE rate. It is very difficult to define the indications for EUS-PD, as pancreatic strictures can be caused by various complicated conditions. The technique, devices, and follow-up protocol of EUS-PD should be established in a future study.

## Figures and Tables

**Table 1:** Patients' characteristics and outcomes of EUS-PD.

Case no.	Sex	Age (years)	Indication	Benign or malignant	Diameter of PD (mm)	Technical success	Clinical success	Rendezvous procedure	Adverse events	Reintervention
1	M	75	Anastomotic stricture after pancreaticojejunostomy	Benign	4	No	NA	NA	None	NA
2	M	67	Anastomotic stricture after pancreaticojejunostomy	Benign	4	No	NA	NA	None	NA
3	M	64	Pancreatic stricture with chronic pancreatitis	Benign	21	Yes	Yes	No	Bleeding	Yes
4	M	70	Anastomotic stricture after pancreaticogastrostomy	Benign	8	Yes	Yes	Yes	Stent migration	No
5	M	47	Pancreatic stricture with chronic pancreatitis	Benign	10	Yes	Yes	Yes	None	Yes
6	M	78	Anastomotic stricture after pancreaticogastrostomy	Benign	18	Yes	Yes	No	None	No
7	F	66	Anastomotic stricture after pancreaticojejunostomy	Benign	6	Yes	Yes	No	None	Yes
8	M	43	Anastomotic stricture after pancreaticojejunostomy	Benign	4	Yes	Yes	No	Peritonitis	Yes
9	M	70	Obstructive pancreatitis with pancreatic cancer	Malignant	7	Yes	Yes	No	None	No
10	F	83	Obstructive pancreatitis with pancreatic cancer	Malignant	9	Yes	Yes	No	None	Yes
11	M	64	Obstructive pancreatitis with pancreatic cancer	Malignant	7	Yes	Yes	No	None	No
12	F	82	Obstructive pancreatitis with pancreatic cancer	Malignant	12	Yes	Yes	No	None	No
13	M	88	Obstructive pancreatitis with pancreatic cancer	Malignant	7	Yes	No	No	None	No
14	M	77	Obstructive pancreatitis with cholangiocarcinoma	Malignant	5	Yes	Yes	No	None	No
15	F	66	Obstructive pancreatitis with pancreatic cancer	Malignant	4	Yes	Yes	No	Peritonitis	No

EUS-PD, endoscopic ultrasound-guided pancreatic duct drainage; PD, pancreatic duct; NA, not available.

**Table 2:** Results of previous studies performed on EUS-PD.

Reference	Study design	Number of patients	Technical success (%)	Clinical success (%)	Adverse events (%)
Tvberg et al. [13]	Prospective observational	80	89	81	20
Fujii et al. [12]	Retrospective	45	74	83	6
Tessier et al. [10]	Retrospective	36	92	70	14
Oh et al. [14]	Prospective observational	25	100	100	20
Ergun et al. [15]	Retrospective	20	90	72	10
Kurihara et al. [16]	Retrospective	17	88	100	6

## Data Availability

The data used to support the findings of this study are available from the corresponding author upon request.
